# COVID‐19 diagnostic testing: Technology perspective

**DOI:** 10.1002/ctm2.158

**Published:** 2020-08-22

**Authors:** Meng Xu, Dan Wang, Hongye Wang, Xiaomei Zhang, Te Liang, Jiayu Dai, Meng Li, Jiahui Zhang, Kai Zhang, Danke Xu, Xiaobo Yu

**Affiliations:** ^1^ State Key Laboratory of Proteomics Beijing Proteome Research Center National Center for Protein Sciences Beijing Institute of Lifeomics Beijing China; ^2^ State Key Laboratory of Analytical Chemistry for Life Science School of Chemistry and Chemical Engineering Nanjing University Nanjing China

**Keywords:** COVID‐19, diagnostic test, nucleic acid, SARS‐COV‐2, serum

## Abstract

The corona virus disease 2019 (COVID‐19) is a highly contagious disease caused by the severe acute respiratory syndrome coronavirus 2 (SARS‐CoV‐2). More than 18 million people were infected with a total of 0.7 million deaths in ∼188 countries. Controlling the spread of SARS‐CoV‐2 is therefore inherently dependent on identifying and isolating infected individuals, especially since COVID‐19 can result in little to no symptoms. Here, we provide a comprehensive review of the different primary technologies used to test for COVID‐19 infection, discuss the advantages and disadvantages of each technology, and highlight the studies that have employed them. We also describe technologies that have the potential to accelerate SARS‐CoV‐2 detection in the future, including digital PCR, CRISPR, and microarray. Finally, remaining challenges in COVID‐19 diagnostic testing are discussed, including (a) the lack of universal standards for diagnostic testing; (b) the identification of appropriate sample collection site(s); (c) the difficulty in performing large population screening; and (d) the limited understanding of SARS‐COV‐2 viral invasion, replication, and transmission.

## Graphical abstract

A comprehensive review of the different primary technologies used to test for COVID‐19 infection. The review indicates the advantages and disadvantages of each technology, describes technologies that have the potential to accelerate SARS‐CoV‐2 detection in the future, and discusses the remaining challenges in COVID‐19 diagnostic testing.

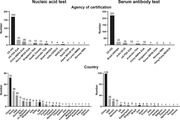

## 1 INTRODUCTION

**FIGURE 1:**
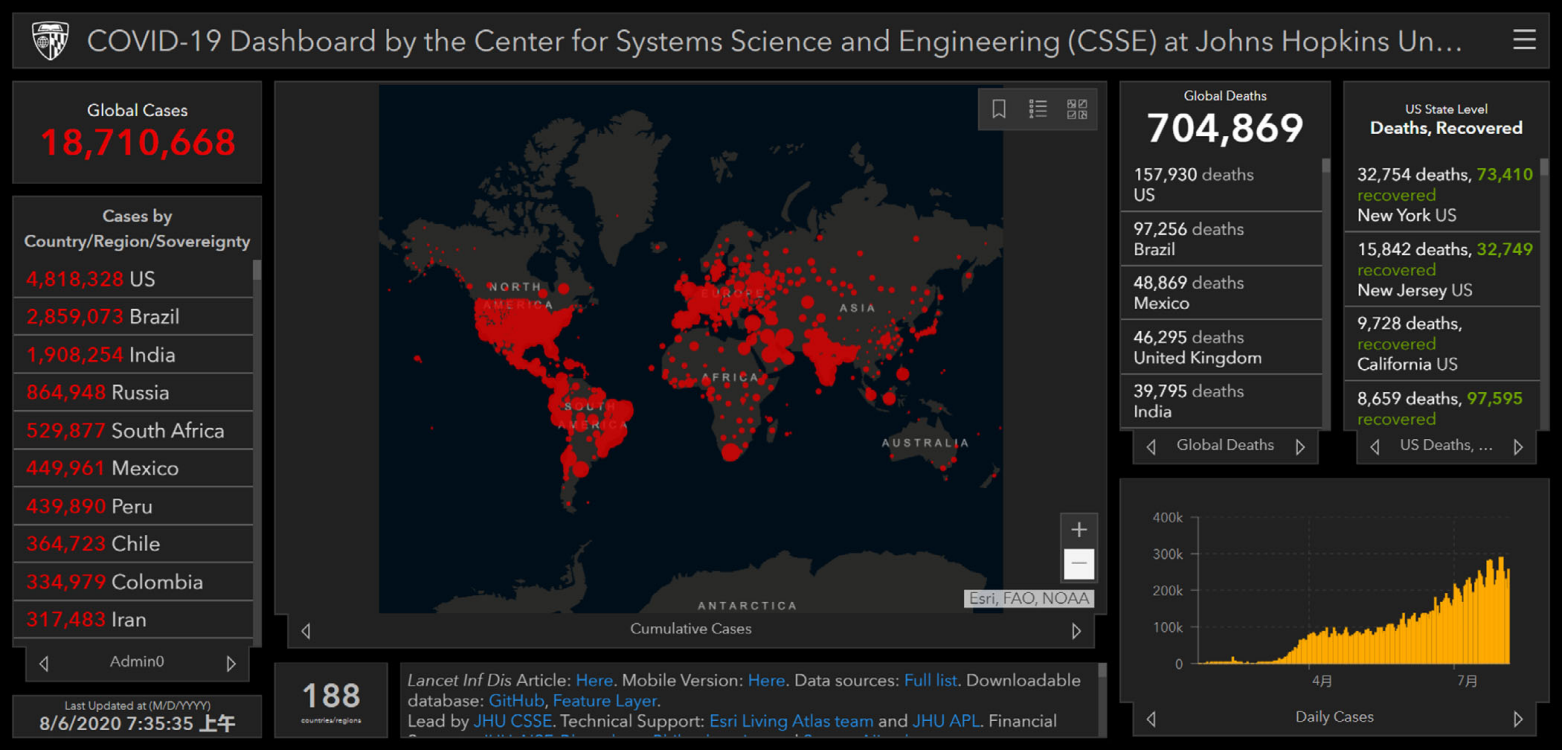
Confirmed COVID‐19 cases as of August 5, 2020. The graph was obtained from an online interactive dashboard developed by the Center for Systems Science and Engineering (CSSE) at Johns Hopkins University, Baltimore, MD, USA^5^

The corona virus disease 2019 (COVID‐19) is a highly contagious disease caused by the severe acute respiratory syndrome coronavirus 2 (SARS‐CoV‐2), and the incubation period is from two to twelve days.^1^, ^2^ Notably, 17.9% of infected individuals who are asymptomatic or mildly symptomatic are still infectious, which has contributed to the rapid worldwide spread of COVID‐19.^3^, ^4^ As of August 5, 2020, over 18 million confirmed cases have been reported in ∼188 countries with ∼0.7 million related deaths (Figure 1).^5^ A standard epidemiological model has predicted that the reproduction number “R0” of SARS‐CoV‐2, which is 2‐3.5, would be reduced to <1.0 by identifying and isolating the majority of infected individuals, including those who might be asymptomatic or mildly symptomatic.^6^, ^7^, ^8^ Thus, the identification and isolation of infected people is paramount in fighting COVID‐19 since vaccines and effective antiviral drugs have not yet been developed.^6^, ^9^

**FIGURE 2:**
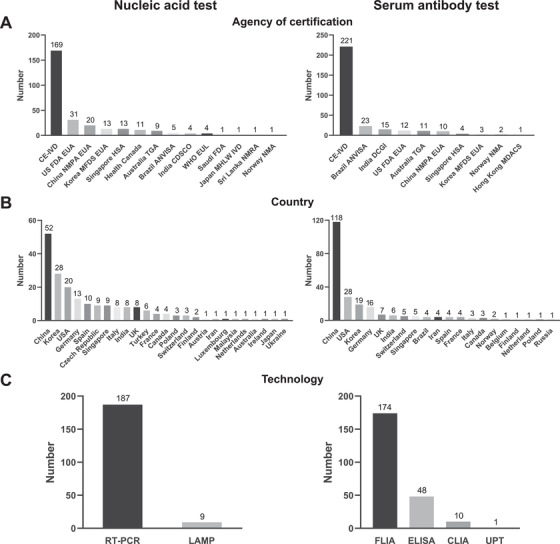
Diagnostic tests that have been approved according by a country's or region's agency of certification. Panels (A‐C) show the nucleic acid and antibody tests that have been approved by different agencies of certification, countries, and technologies, respectively. The data were obtained from the FIND COVID‐19 diagnostics resource center (https://www.finddx.org/covid-19/) on June 9, 2020. Abbreviations: EUA, Emergency Use Authorization; HSA, Health & Safety/Sciences Authority; MFDS, Ministry of Food & Drug Safety; MHRA, Medicines & Health Care Products Regulatory Agency; NRA, National Regulatory Authority; RUO, Research Use Only; TGA, Therapeutic Goods Administration; WHO EUL, World Health Organization Emergency Use Listing Procedure

Tremendous efforts have been employed to develop highly accurate diagnostic testing for COVID‐19 since January 2020.^10^ Together, the World Health Organization (WHO) and Foundation for Innovative New Diagnostics (FIND) are collaborating to independently validating tests from different manufactures across the globe.^10^, ^11^ The U.S. Food and Drug Administration (FDA) initiated fast‐tracked FDA review and approval of COVID‐19 tests through emergency use authorization (EUA) in mid‐March.^11^ According to the data from FIND COVID‐19 resource center (https://www.finddx.org/covid-19/) on June 9, 2020, over 400 molecular and serological antibody tests have been approved by different countries’ and regions’ agencies of certification. Among them, 196 molecular tests and 233 serum tests were approved by Conformité Européenne (CE), followed by the US FDA, China National Medical Products Administration (NMPA), and Brazil Brazilian Health Regulatory Agency (ANVISA) (Figure 2A and 2C). Most of the molecular (M) and serum (S) tests for COVID‐19 detection have been produced by China (52 M, 118 S), followed by the USA (20 M, 28 S), Korea (28 M, 19 S), and Germany (13 M, 16 S) (Figure 2B and 2C).

The type of technologies that a test is based on has a significant impact on the test's diagnostic performance, including sensitivity, specificity, dynamic range, reproducibility, reagent consumption, equipment, cost, throughput, and ease‐of‐use. For example, the successful identification of SARS‐CoV‐2 is attributed to metagenomics next‐generation sequencing (mNGS) technology.^12^ SARS‐CoV‐2 nucleic acid testing enables the early COVID‐19 patients detection whereas acute and recovered patients can be detected with antibody tests.^13^, ^14^

Here, a comprehensive review of the major COVID‐19 diagnostic tests is provided, including their unique advantages and disadvantages. Promising technologies in SARS‐CoV‐2 detection are also discussed. The data were obtained by searching PubMed, Preprint servers (ie, medRxiv, bioRxiv), and related databases for English‐based articles with terms such as “SARS‐CoV‐2,” “COVID‐19,” “nucleic acid testing,” “serum testing,” “diagnostics,” or their lexical variants since January 1, 2020.

## 2 TECHNOLOGY ON THE COVID‐19 DIAGNOSTIC TESTING

### 2.1 Molecular assay

**FIGURE 3:**
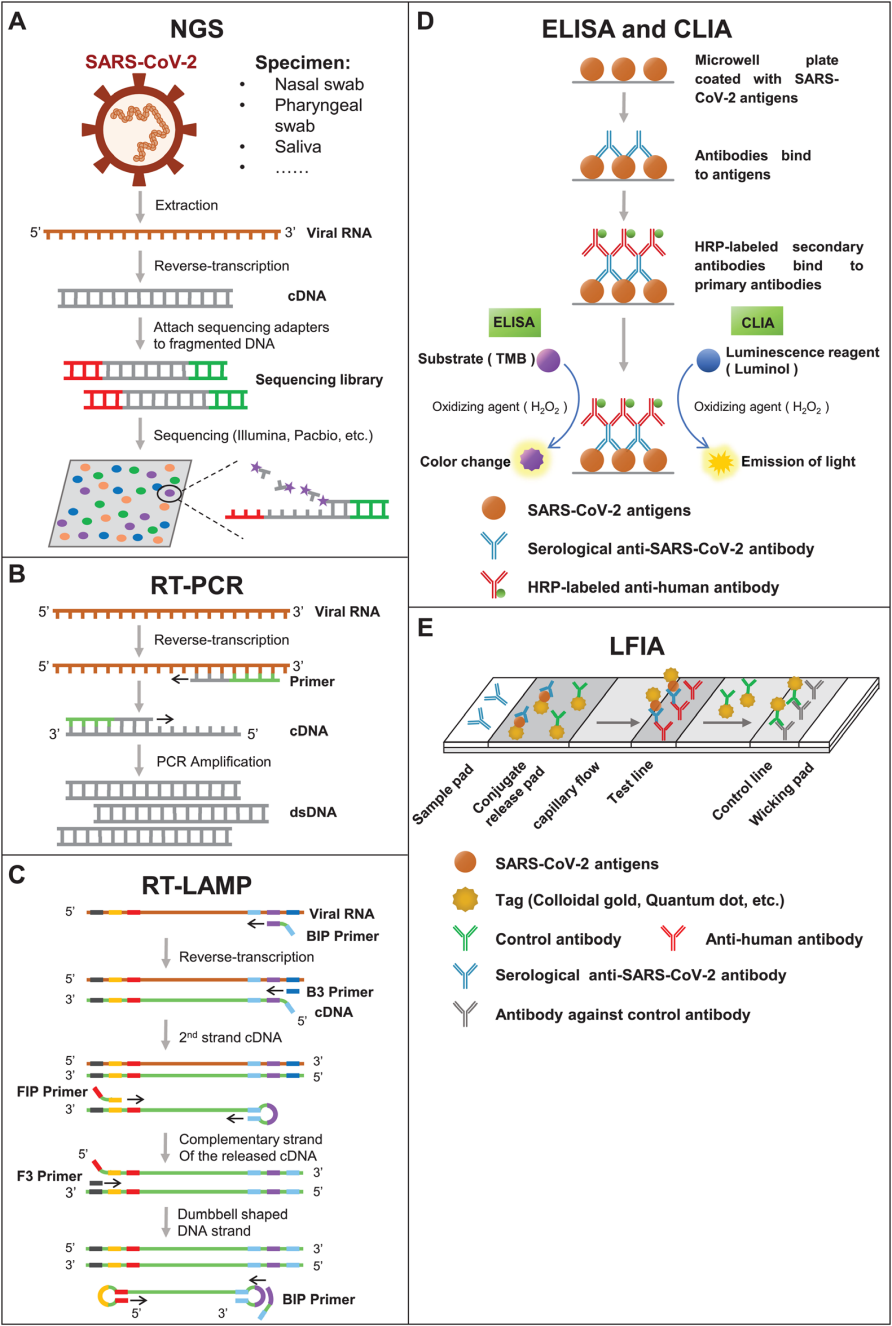
Schematic illustration of nucleic acid and serum testing methods. Panels (A‐E) are the assay principles of NGS, RT‐PCR, RT‐LAMP, ELISA, CLIA, and LFIA, respectively

A molecular assay tests for the presence of specific genetic material (eg, viral RNA) in a biological specimen. As of June 9, 2020, over ∼200 molecular tests have been approved for in vitro diagnostics of COVID‐19, which include Next‐Generation Sequencing (NGS),^15^ reverse transcription polymerase chain reaction (RT‐PCR),^16^, ^17^, ^18^ and loop‐mediated isothermal amplification (LAMP)^19^ (Figure 3).

#### 2.1.1 Next‐generation sequencing

NGS is a genomics technology that enables the simultaneous sequencing of thousands to billions of DNA fragments, and can identify genetic material from entirely different kingdoms of organisms (Figure 3A).^20^ Applications of NGS technology include diagnosing infectious diseases, tracking outbreaks, infection control surveillance, and mutation and pathogen discovery^21^ with different types of biological specimens (ie, cerebrospinal fluid, blood, respiratory samples, gastrointestinal fluid, and ocular fluid).^22^, ^23^ Sequencing platforms are available from Illumina (iSeq, MiSeq, MiniSeq, NextSeq, HiSeq, NovaSeq), Thermo Fisher Scientific (Ion Torrent), BGI (BGISEQ platform), and Oxford Nanopore Technologies (MinION, GridION, PromethION).^24^

SARS‐CoV‐2 was first isolated as an unknown virus in five COVID‐19 patients, and then identified in two COVID‐19 patients using metagenomics NGS (mNGS).^12^, ^15^ The sequence of this virus is 79.6% identical to the virus responsible for the 2003 SARS outbreak, SARS‐CoV, and 96% to a bat coronavirus.^12^ Phylogenetic analyses of the SARS‐CoV‐2 virus that was extracted from the bronchoalveolar lavage fluid and cultured isolates in nine COVID‐19 patients categorized SARS‐CoV‐2 as subgenus *Sarbecovirus* of the genus *Betacoronavirus*, with its closest viral relatives being the bat‐SL‐CoVZC45 and bat‐SL‐CoVZXC21.^25^ Notably, the SARS‐CoV spike (S) receptor binding domain (RBD) that interacts with the human host receptor, ACE2, is very different from RBD of SARS‐CoV‐2.^26^, ^27^ These mutations result in a different binding affinity between the S protein and ACE2. Over 18 000 SARS‐CoV‐2 sequences have thus far been collected and stored in the GISAID database (https://www.gisaid.org/).

However, the analytical sensitivity in pathogen detection is limited with NGS because microbial nucleic acids from resident microbiota often result in high background. In fact, >99% of the reads are often from the human host.^24^ Large‐scale population screening is limited by the high cost to perform NGS.

#### 2.1.2 RT‐PCR

Using RT‐PCR, the viral RNA can be reversely transcribed into complementary DNA that is then amplified using directed primers that flank the DNA sequence‐of‐interest (Figure 3B). RT‐PCR is fast, yet also has high specificity and simple quantification; it is considered the gold standard in SARS‐CoV‐2 diagnosis.^28^, ^29^, ^30^, ^31^ Briefly, swabs of the back of the throat or nasal cavity are taken, saliva can also be used. RNA is extracted, and then RT‐PCR is performed. Padhye et al evaluated the performance of RT‐PCR with data in 1014 patients, including 601 COVID‐19 positive and 413 healthy controls. The sensitivity and specificity of the RT‐PCR detection was estimated to be 0.777 (95% confidence interval (CI): 0.715‐0.849) and 0.988 (95% CI: 0.933‐1.000), respectively.^32^

False negatives can occur with RT‐PCR testing if the viral copies are too low to be amplified. Viral copy varies across individuals, specimen type (ie, nasal, throat or sputum) and days post infection.^16^, ^33^, ^34^, ^35^, ^36^ Another limitation of RT‐PCR is that the assay must be performed in a Clinical Laboratory Improvement Amendments of 1988 (CLIA) accredited laboratory with appropriate equipment and experienced operators.^11^ The process, from RNA extraction to RT‐PCR to data analysis, can be completed in ∼1 day.

#### 2.1.3 RT‐LAMP

Comparing to RT‐PCR, RT‐LAMP does not require a PCR machine and thus the assay is rapid and inexpensive. Briefly, primers, reverse‐transcriptase, Bst DNA polymerase, and a pH indicator dye are mixed with extracted sample RNA and then heated to 65˚C for 60 min (Figure 3C).^37^ As the DNA is amplified, the pH decreases, causing a colorimetric change that is visualized by eye. Also, the primers are specially designed so that they “loop” or anneal onto each other to enable rapid DNA amplification without the need for thermal cycling, thus making the RT‐LAMP method faster than RT‐PCR. Unlike RT‐PCR, RT‐LAMP is suitable for on‐site detection because it has high specificity, is simple to perform, cost effective, and only requires a heating block.^38^, ^39^

Jiang et al developed a RT‐LAMP method using primers targeting SARS‐CoV‐2 genes encoding for the ORF1ab, envelope (E), and nucleocapsid (N) proteins. The sensitivity of 91.4% and the specificity of 99.50% were obtained from 47 and 213 patients with and without SARS‐CoV‐2 infection.^38^ Similar results were obtained in an independent study with 17 and 191 patients who had or did not have COVID‐19, respectively.^40^ A major drawback of RT‐LAMP is that it is low throughput because only one sample can be measured in an experiment.

### 2.2 Immunological assay

The detection of SARS‐CoV‐2 antibodies is helpful in identifying prior infection and immunity, which is of significance to epidemiologic and vaccine studies, ongoing surveillance and to evaluate the risk of health care workers.^11^, ^41^, ^42^, ^43^ Notably, COVID‐19 antibody profiles across time and the role of IgG in immunity are still being ascertained. Recent studies indicate that the SARS‐CoV‐2 IgM antibodies start ∼7 days after symptom onset and peak at day 28, while IgG start ∼10 days after infection and peak at day 49.^41^ However, antibody profiles vary across individuals. Furthermore, the expression of IgM and IgG antibodies are higher in COVID‐19 patients with severe symptoms than patients with milder symptoms (*P* < .05). COVID‐19 patients with low antibody responses tend to have a higher viral clearance rate than patients with stronger antibody responses (*P* = .011).^44^, ^45^ Over 190 COVID‐19 serology tests have been approved by agencies of certification according to the FIND database. These tests include the enzyme‐linked immune sorbent assay (ELISA), chemiluminescence enzyme immunoassay (CLIA), and lateral flow immunochromatographic assay (LFIA) (Figure 3).^46^, ^47^

#### 2.2.1 Enzyme‐linked immunosorbent assay

As a qualitative or semi‐quantitative detection method, indirect ELISA immobilizes the antigen‐of‐interest on a solid substrate (eg, 96‐well plate) to capture its specific antibody. Antibody binding is often detected using an enzyme‐labeled, secondary antibody that produces a color change in the presence of an enzyme‐catalyzed substrate, which is subsequently detected using a common laboratory instrument (ie, plate reader) (Figures 3D). The advantage of ELISA assay is easy to operate and low cost. It can also be easily made high throughput with an automated workstation.

Zhao et al screened antibodies in the serum of 173 COVID‐19 patients using an indirect ELISA where the S protein's RBD was immobilized onto the plate. The total, IgM, and IgG antibodies were observed in 93.1% (161/173), 82.7% (143/173), and 64.7% (112/173) of COVID‐19 patients, respectively. The median times for seroconversion of total, IgM, and IgG antibodies is 11, 12, and 14 days, respectively. Furthermore, detecting both viral RNA and antibodies can significantly improve the diagnostic sensitivity for COVID‐19 (*P* < .001), especially for the early viral infection (<7 days following symptom onset) (*P* = .007).^44^

#### 2.2.2 CLIA

CLIA is similar to ELISA, except that antibody binding to the immobilized substrate is detected via a change in luminescence (Figure 3D). More specifically, a different enzyme catalyzed substrate is used for CLIA (ie, luminol) than ELISA (ie, 3,3′,5,5′‐tetramethylbenzidine). The CLIA assay has higher sensitivity than ELISA and has been fully automated, such that hundreds of samples can be screened easily within a day.^48^

Using CLIA, Long et al first performed a multi‐center cross‐sectional analyses of 285 patients to determine the diagnostic value of SARS‐CoV‐2 IgM and lgG antibodies. He then analyzed 63 patients in a single‐center follow‐up study. The positive rate of IgM and IgG antibodies reached 94.1% and 100% on days 20‐22 and 17‐19 after symptom onset, respectively. In addition, four and seven patients displaying COVID‐19 symptoms who obtained false negative results with RT‐PCR were confirmed to have COVID‐19 based on their antibody response using CLIA.^49^ Similar results were obtained by Zhang et al who analyzed 736 COVID‐19, non‐COVID‐19, or healthy patients.^50^ Here, areas under the receiver operating characteristic (ROC) curves for IgM and IgG of 0.988 and 1.000 were obtained, respectively.

Of note, the total, IgM, IgG, and IgA antibodies to SARS‐CoV‐2 were also detected by Zhang et al in 56 asymptomatic patients, in which 23 patients were keeping asymptomatic in the follow‐up period. Nucleic acid testing indicated that the SARS‐CoV‐2 replication in nasopharyngeal cavity were no difference among asymptomatic carriers, pre‐symptomatic, and symptomatic patients. Only IgM antibodies had an obvious difference in the seroconversion rate among the three groups. Interestingly, the asymptomatic patients had low levels of IgM antibodies and high total IgG and IgA compared to symptomatic patients. These results might be helpful to understand viral clearance and antibody changes in COVID‐19 patients of asymptomatic.^51^

#### 2.2.3 LFIA

LFIA is a qualitative point‐of‐care (POC) testing device which can detect SARS‐CoV‐2 antibodies within 5‐30 min in urine, saliva, sweat, serum, plasma, whole blood, and other fluids.^52^ First, a SARS‐CoV‐2 antigen(s) is immobilized onto the middle of a strip, which is often a nitrocellulose membrane. Then, samples are applied to one end of a strip where Colloidal gold (CG) or quantum dot (QD)‐labeled detection antibodies are present. Then, the sample and labeled antibodies will migrate along the strip. If SARS‐CoV‐2 antibodies are present, they will be bound by the labeled detection antibody and will bind to the immobilized antigen. The immobilized antibody‐detection antibody complex will result in the appearance of a band on the strip due to the accumulation of CG or QD^53^ (Figure 3E).

**FIGURE 4:**
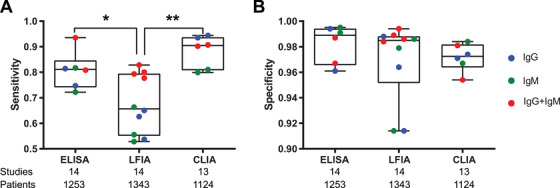
Comparison of different technologies used for serological antibody detection. The data was obtained from the Kontou study by meta‐analysis of antibody testing in 7848 individuals.^54^

LFIA is simple to use (like a home pregnancy test), cost effective, can be used for onsite detection, and does not require an instrument. The assay performance of nine different LFIA devices was determined by screening 40 RT‐PCR‐positive COVID‐19 individuals and 142 negative controls collected prior to December 2019 from the Biobank in the United Kingdom. The systematic, meta‐analysis comparison of ELISA, CLIA, and LFIA was performed based on 38 studies with 7848 individuals. Regardless of the antigens whose detail is normally not released by the manufactures, the serum testing using ELISA and CLIA‐based methods had higher sensitivity (90% to 94%) than LFIA (Figure 4).^54^ These results demonstrate that the serum testing using ELISA and CLIA are more reliable, while the LFIA results should be considered with caution.^43^

LFIA is not only used to detect SARS‐CoV‐2 antibodies, but also to detect SARS‐CoV‐2 proteins in clinical specimens. Here, the detection antibody will be specific to the antigen‐of‐interest. However, only one COVID‐19 test that detects SARS‐CoV‐2 proteins (Sofia 2 SARS Antigen FIA) has been approved to date. The point‐of‐care testing must be performed in a certified laboratory under a CLIA Certificate of Waiver. We recently screened the serum of 8 COVID‐19 patients with mild symptoms using a sandwich ELISA developed in our laboratory with a lower detection limit of 200 pg/mL (data not shown).^55^ However, none of the samples were positively identified, which is likely because serum has low copies of SARS‐CoV‐2 virus.^47^ The levels of SARS‐CoV‐2 proteins in different sampling locations across the stages of COVID‐19 infection should be examined.^56^

## 3 PROMISING TECHNOLOGIES FOR COVID‐19 DETECTION

**FIGURE 5:**
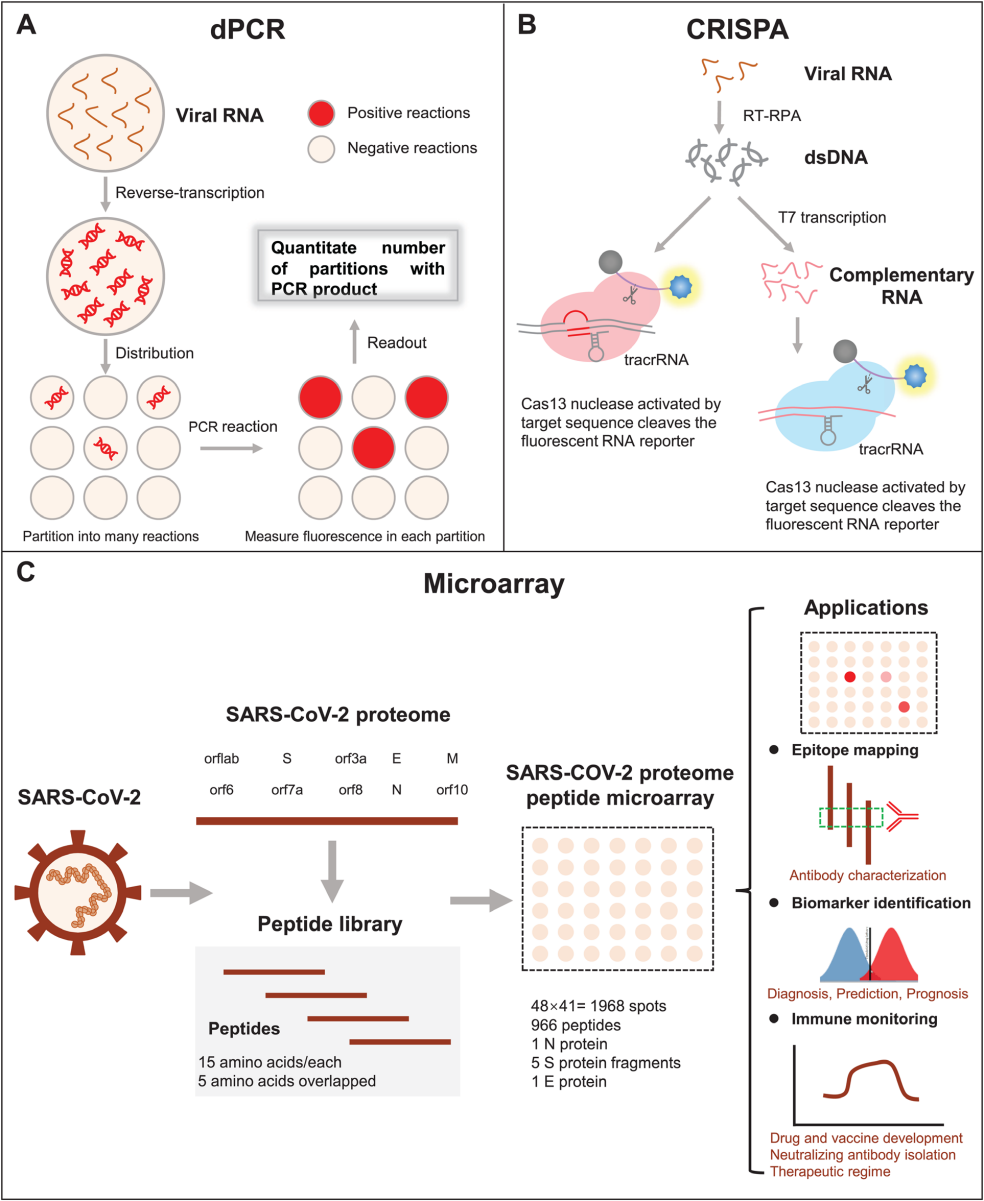
Schematic illustration of promising technologies for SARS‐CoV‐2 detection. (A‐C) are the assay principles of dPCR, CRISPR, and SARS‐CoV‐2 proteome peptide microarray, respectively

Asymptomatic and mildly symptomatic individuals are rarely tested for COVID‐19, which is partly because current technologies are limited in throughput. To address this challenge, different technologies other than those described above have either been developed or are in development to detect SARS‐CoV‐2 in high throughput with high sensitivity.^57^, ^58^, ^59^, ^60^ Here, we review three such technologies: digital PCR (dPCR), Clustered regularly interspaced short palindromic repeats (CRISPR), and SARS‐CoV‐2 proteome peptide microarray (Figure 5).

### 3.1 dPCR

dPCR partitions nucleic acid molecules into tens of thousands to hundreds of thousands of reaction units. Each reaction unit carries out PCR amplification independently. Then, the fluorescence signal of each reaction chamber was analyzed, and the copy number of target nucleic acid sequence was calculated and quantified^61^ (Figure 5A). According to the type of liquid separation, dPCR can be divided into microfluidic digital PCR (mdPCR), droplet digital PCR (ddPCR), and chip digital PCR (cdPCR). An Important advantage of dPCR is that the quantification is independent of variations in the amplification efficiency.^62^ In addition, it does not require internal reference genes, nor does it need a standard curve, which is more tolerant to interference factors such as specific template amplification inhibitors, and can achieve accurate quantification of low concentration nucleic acid samples. dPCR is used to analyze viral load and gene copy number variation.^63^, ^64^, ^65^, ^66^, ^67^

Using RT‐dPCR, Suo et al detected 57 pharyngeal swab samples for the presence of SARS‐CoV‐2. An overall accuracy of 94.3% was obtained, ∼500 times more sensitive than RT‐PCR.^68^ Lu et al compared the RT‐dPCR and RT‐PCR with 108 specimens (pharyngeal swab, stool, and blood) longitudinally collected from 36 COVID‐19 patients. The RT‐dPCR showed the detection limit 10× lower than that of RT‐PCR. Notably, four pharyngeal swab samples showed negative in RT‐PCR testing were positive using RT‐dPCR.^69^ Dong et al used RT‐dPCR to measure 194 pharyngeal swab samples from close contacts, suspected, and recovered COVID‐19 patients. The sensitivity of 90% and the specificity of 100% were obtained. Comparing to RT‐PCR sensitivity (28.2%), the RT‐dPCR showed the improved sensitivity of 87.4% in the detection of 103 suspects.^70^

### 3.2 CRISPR

In 2013, CRISPR/Cas technology developed rapidly as a third generation genome editing technology.^71^, ^72^ Briefly, the CRISPR/Cas system captures and inserts foreign DNA fragments into the bacterial genome, and then guides the Cas endonuclease to remove the foreign nucleic acid (Figure 5B). The CRISPR/Cas technology is characterized by high specificity, high precision, high efficiency, and simple and fast operation. Broughton et al developed an accurate CRISPR‐Cas12‐based lateral flow assay that can easily detect the SARS‐CoV‐2 N and E genes in RNA extracts from respiratory swabs within 40 min. The method was validated using 36 COVID‐19 patients and 42 patients infected with different respiratory viruses,^73^ offering a promising poTable POC testing for SARS‐CoV‐2 screening.^13^ On May 7, 2020, the US FDA granted its first EUA coronavirus test using CRISPR technology.^74^

### 3.3 Microarray

Microarray technology enables the high‐sensitive detection of hundreds or even thousands of molecules simultaneously using a low sample volume^75^, ^76^, ^77^ (Figure 5C). Our team developed a SARS‐CoV‐2 proteome microarray to detect hundreds of antigen‐antibody interactions in serum at amino acid resolution within 1.5 h. The array has SARS‐COV‐2 N and E full‐length proteins, and five S truncated proteins as well as 966 tiled peptides covering all amino acid sequences of SARS‐CoV‐2 proteins. Using the array, we identified immunogenic regions of the SARS‐CoV‐2 proteins and an epitope for potential neutralizing antibodies to viral entry (ie, the interaction between the S protein's RBD and the human receptor, ACE2).^55^, ^78^ Similar arrays were developed by Dahlke et al^79^ using in situ synthesized peptides and Jiang et al^80^ and Assis et al^81^ using purified SARS‐CoV‐2 antigens. The comprehensive examination of humoral antibody responses in COVID‐19 patients using protein microarrays would be valuable in understanding immunity, identifying diagnostic targets, and developing COVID‐19 therapies.

## 4 PROSPECTIVE

Every COVID‐19 diagnostic test has its advantages and limitations (Tables 1 and 2), and there remain several key challenges for all of them. These challenges, which are described in more details below, include the lack of a standard in COVID‐19 diagnostic testing, difficulty in identifying asymptomatic or mildly symptomatic patients as early as possible, and limited understanding of the virus and disease pathology:

**TABLE 1:** Advantages and disadvantages of the different technologies used to detect SARS‐CoV‐2 viral RNA

Technology	NGS	RT‐PCR	RT‐LAMP
Detection time	0.5‐3 days^38^, ^59^	0.5‐1 h^29^, ^111^, ^112^	0.5‐1 h^38^, ^40^, ^113^
Sensitivity	N. A.^†^	73%‐86%^32^, ^33^, ^114^	81% ‐ 100% ^38^, ^40^, ^115^
Specificity	100%^116^	86% ‐ 100% ^32^, ^33^, ^114^	99% ‐ 100%^38^, ^40^, ^115^
Advantages	Unknown virus identification, viral mutation and evolution	High sensitivity and specificity	On‐site detection, simple operation, cost effective
Disadvantage	Long turnaround time, expensive	Sophisticated equipment, experienced operators	Low throughput
Application	Diagnostics,^117^ lineage tracing^15^ and mutation discovery,^118^ infection control surveillance^119^	Diagnostics^120^, epidemiological surveillance^121^, discharge from hospital^16^	Diagnostics,^39^ Epidemiological surveillance^122^

^†^ N. A., not available.

**TABLE 2:** Advantages and disadvantages of the different technologies used to detect COVID‐19 antibodies

Technology	ELISA	CLIA	LFIA
Detection time	1.5‐2.5 h^123^	0.5 h ^50^	10‐15 min^57^, ^124^
Sensitivity	65‐98%^123^, ^125^, ^126^, ^127^, ^128^	77‐100% ^114^, ^125^, ^127^, ^129^	69‐93%^123^, ^124^, ^125^, ^127^, ^130^
Specificity	71‐100%^123^, ^125^, ^126^, ^127^, ^128^	90‐100% ^114^, ^125^, ^127^, ^129^	80‐100%^123^, ^124^, ^125^, ^127^, ^130^
Advantages	Simple operation, low cost, high‐throughput	Higher sensitivity and specificity, broad linearity, automated	Simple operation, low cost, rapid
Disadvantage	Time‐consuming, Vulnerable to contamination	Need Supporting chemiluminescence instruments	Low sensitivity during early SARS‐COV‐2 infection
Application	Diagnostics,^131^ epidemiological surveillance,^132^ discharge from hospital^133^	Diagnostics^49^, epidemiological surveillance^50^, discharge from hospital	Diagnostics,^124^ Epidemiological surveillance^134^

**1) No universal standard for diagnostic testing**. A universal standard is critical in generating consistent tests by different manufacturers and enables laboratories worldwide to compare results easily.^11^, ^56^, ^82^ For example, there are at least seven versions of PCR primers used in SARS‐CoV‐2 nucleic acid testing (Table 3).^11^, ^83^ The US Centers for Disease Control (CDC) recommend primers targeting two regions of the N gene. China's CDC recommend primers targeting the ORF1ab and N gene, and France's Institut Pasteur recommends primers targeting the RdRP gene. Similarly, different SARS‐CoV‐2 proteins or protein fragments are employed in immunological testing.^54^ To address this issue, WHO, FIND, and other research laboratories are collaborating with each other or working independently to evaluate the performance of different diagnostic tests across the world. For example, Jung et al compared seven primer probe sets for the N gene and the three primer‐probe sets for the Orf1 gene to detect SARS‐CoV‐2 viral RNA. The result showed that the most sensitive primer‐probe sets of N and Orf1 genes are ‘2019‐nCoV_N2, N3’ from the U.S. CDC and the ‘ORF1ab’ from China CDC, respectively.^84^

**TABLE 3:** Gene targets recommended for nucleic acid testing by different countries and regions‡

Institute	Gene targets
China CDC, China	ORF1ab and N
Institut Pasteur, France	Two targets in RdRP
US CDC, USA	Three targets in N gene
National Institute of Infectious Diseases, Japan	Pancorona and multiple targets, Spike protein
Charité, German	RdRP, E, N
HKU, Hong Kong SAR	ORF1b‐nsp14
National Institute of Health, Thailan	N

^‡^The data were obtained from the Coronavirus disease (COVID‐19) technical guidance: Laboratory testing for 2019‐nCoV in humans in WHO (https://www.who.int/emergencies/diseases/novel-coronavirus-2019/technical-guidance/laboratory-guidance).

We measured the SARS‐CoV‐2 antibodies in early COVID‐19, influenza, and non‐influenza patients using the microarray described above.^55^ We found that the N protein might not be an ideal antigen for early SARS‐CoV‐2 testing because the similar IgM antibody profiles was found in COVID‐19 patients and influenza patients. Instead, the RBD and extracellular domain (ECD) from SARS‐CoV‐2 S protein could be a better option for IgM antibody detection, whereas the ECD is more suitable for IgG detection.^85^ The results can be also supported by a meta‐analysis of 7848 individuals, in which the S antigen displayed superior sensitivity (81.4% IgG, 81.7% IgM) compared to the N antigen (74.7% IgG, 72.2% IgM) using ELISA.^54^, ^77^ We speculate that the reason why more antibodies are developed to the S protein is because the S protein is on the SAR‐CoV‐2 surface and is more easily recognized by the immune system.^80^, ^85^

**2) Standardization of sample collection**. The specimen and collection time from infected patient may have great influence on the detection result.^30^, ^86^, ^87^ Currently nasal swabs and pharyngeal swabs are the major clinical samples used for nucleic acid testing. However, collection of nasopharyngeal specimens is uncomfortable for the patients and increases the risk of infection to health‐care workers. Wang et al used RT‐PCR to detect the SARS‐CoV‐2 virus in different types of clinical samples. The positive rate of broncho alveolar lavage fluid, sputum, nasal swabs, fibro bronchoscope brush biopsy, pharyngeal swabs, feces, and blood are 93% (14 of 15), 72% (72 of 104), 63% (5 of 8), 46% (6 of 13), 32% (126 of 398), 29% (44 of 153), and 1%, respectively. Whereas, none positive was detected in 72 urine specimens.^86^ Using RT‐PCR, To et al detected the SARS‐CoV‐2 S gene in the saliva of 11 out of 12 COVID‐19 patients.^88^ In another work, the same group found that the viral load in saliva reached the peak at 7 days of symptom onset and then decreased, indicating the potential of using saliva for diagnosis and viral load monitoring.^89^, ^90^, ^91^ On April 14, 2020, The US FDA authorized the first saliva test for emergency use in COVID‐19 diagnosis, which was developed by Rutgers’ RUCDR Infinite Biologics in partnership with Spectrum Solutions and Accurate Diagnostic Labs (ADL).

Exosomes are small membrane vesicles (40‐100 nm in size) with the potential to transfer complex RNAs and proteins between cells,^92^ which had been demonstrated to be a promising specimen for the detection of lung cancer, breast cancer, bladder cancer, and other critical illness.^93^, ^94^, ^95^, ^96^, ^97^ Recently, it was found that exosomes can transfer ACE2 to receipt cells supporting the SARS‐CoV‐2 internalization and infection,^98^ indicating the relationship between exosomes and COVID‐19 pathogenesis.^99^ However, the diagnostic value of exosome in COVID‐19 has yet to be determined.

**3) Identification of asymptomatic and mildly symptomatic patients require large population screening**. Identifying and quarantining infected people – including those who are asymptomatic and mildly symptomatic – at home or shelter is important in stopping the spread of COVID‐19.^100^ To do so, large population screening is necessary; thus the assay must be high throughput, rapid, and cheap.^6^, ^10^, ^51^, ^101^, ^102^ For example, Bendavid et al used an LIFA to test the prevalence of SARS‐CoV‐2 antibodies in 3330 people from Santa Clara County, California. The results indicated that 2.49% (95% CI: 1.80‐3.17%) to 4.16% (95% CI: 2.58‐5.70%) people in Santa Clara might be SARS‐CoV‐2 infected.^103^ In another study, Stringhini et al detected IgG antibodies in 1335 participants from Geneva, Switzerland using ELISA. The results show that the seroprevalence increased from 3.1% (95% CI: 0.2‐5.99, n = 343) in the first week to 6.1% (95% CI: 2.6‐9.33, n = 416) and 9.7% (95% CI: 6.1‐13.11, n = 576) in the second and third weeks, respectively.^104^

**4) Limited understanding of viral invasion, replication, and evolution** ^105^, ^106^, ^107^. Accurate COVID‐19 diagnosis and effective treatment will likely depend on better understanding the SARS‐CoV‐2 virus and COVID‐19 pathology. Unfortunately, there are still many unanswered questions. For example, Yao et al recently found that the SARS‐CoV‐2 virus was still present in pneumocytes during a pathological examination of a recovered COVID‐19 patient who passed away due to sudden cardiovascular incident.^108^ These results might explain why some recovered COVID‐19 patients end up having positive PCR results after being discharged from the hospital.^16^

Here, we discussed the numerous types of technologies available to test for COVID‐19, each with their own advantages and disadvantages. It is clear that a global collaboration is urgently needed.^100^, ^109^, ^110^ There are no standard RT‐PCR primers nor universal SARS‐CoV‐2 antigens for antibody detection. Consequently, diagnostic accuracies vary, and test results cannot be easily compared with each other for meta‐analyses that can help shed light on SARS‐CoV‐2 infection and the host immune response (eg, antibody profiles across time). Finally, individuals displaying COVID‐19 symptoms are tested for COVID‐19. However, large‐scale population screening is necessary since infected individuals who are asymptomatic or mildly symptomatic are still contagious. The global exchange of information about patient data, testing data, and molecular studies will be the most rapid approach to identify the most accurate RT‐PCR approach, immunoassay test, and effective therapeutic regimes to stop the spread of COVID‐19.

## AUTHOR CONTRIBUTIONS

M. X., D. W., H. W., X. Z., T. L., J. D., M. L., J. Z., and K. Z., searched the literature. M. X., D. X., and X. Y. prepared the manuscript.

## COONFLICT OF INTEREST

The authors have declared no conflict of interest.
